# A novel combined RNA-protein interaction analysis distinguishes HIV-1 Gag protein binding sites from structural change in the viral RNA leader

**DOI:** 10.1038/srep14369

**Published:** 2015-10-09

**Authors:** Julia C. Kenyon, Liam J. Prestwood, Andrew M. L. Lever

**Affiliations:** 1 Box 157, Cambridge University Dept. of Medicine, Addenbrooke’s Hospital, Hills Rd, Cambridge CB2 0QQ.

## Abstract

RNA-protein interactions govern many viral and host cell processes. Conventional ‘footprinting’ to examine RNA-protein complex formation often cannot distinguish between sites of RNA-protein interaction and sites of RNA structural remodelling. We have developed a novel technique combining photo crosslinking with RNA 2′ hydroxyl reactivity (‘SHAPE’) that achieves rapid and hitherto unachievable resolution of both RNA structural changes and the sites of protein interaction within an RNA-protein complex. ‘XL-SHAPE’ was validated using well-characterized viral RNA-protein interactions: HIV-1 Tat/TAR and bacteriophage MS2 RNA/Coat Binding Protein. It was then used to map HIV-1 Gag protein interactions on 2D and 3D models of the viral RNA leader. Distinct Gag binding sites were identified on exposed RNA surfaces corresponding to regions identified by mutagenesis as important for genome packaging. This widely applicable technique has revealed a first view of the stoichiometry and structure of the initial complex formed when HIV captures its genome.

RNA molecules, like proteins, can adopt intricate three-dimensional structures^1^. These form functional scaffolds for interaction with other RNAs or proteins and can be static, or can switch conformation, sometimes radically, upon ligand binding^2^. RNA structures and their interactions with proteins control many processes in normal cell function and in disease, including transcription, splicing, nuclear export, RNA turnover, translation, transport, and also many viral processes. The structures of RNA molecules are generally less well elucidated than their protein counterparts. How they interact with their ligands is also less well understood than protein-protein interactions, largely because of the structural plasticity of RNA molecules that can be thousands of nucleotides long. X-ray crystallography and NMR are only capable of interrogating smaller, more static structures. Several methods are currently used to footprint the specific binding sites of proteins on their RNA targets^3^,^4^. Mostly these examine the accessibility and reactivity of the RNA molecule on the assumption that at sites where either or both of these diminish a protein is bound. This risks producing a misleading or incomplete picture. As an example, a protein binding to one strand of a helix and displacing the complementary strand to bind elsewhere, could have no apparent footprint upon the helix it interacts with, while the distant RNA to which the displaced helix strand binds shows decreased reactivity mimicking a protein binding site. As evidence for the importance of RNA-protein interactions and their emergence as therapeutic targets increases, a pressing need is for improvements in the arsenal of techniques to study them^5^. We hypothesized that combining a powerful secondary structure probing method (SHAPE- selective 2′OH acylation analyzed by primer extension) with a cross-linking technique would provide a more comprehensive picture of RNA-protein interactions.

We used this technique to gain new insight into HIV-1 genome packaging. HIV-1 is a global pathogen, infecting 35 million people worldwide and causing 7,000 new infections a day^6^. The viral genome is a single-stranded RNA molecule that dimerizes via a palindromic site in the 5′ leader^7^,^8^. This region, along with the beginning of the *gag* gene, is known as the packaging signal (*psi*)^9^,^10^. The viral genome is captured for packaging by the structural protein Gag, in a highly specific process whereby Gag differentiates it from the far more abundant cellular and spliced viral RNAs in the cytoplasm. Although one stem-loop is known to bind Gag with high affinity (SL3), the initial capture is believed to involve a small number of Gag proteins binding to *psi*^11^. Analyzing this interaction is complicated because the RNA changes structure upon dimerization, exposing different regions that are potential additional protein binding sites^12^,^13^. Previous studies using a range of footprinting methods have identified several sites of interaction on the *psi* RNA^14^,^15^. XL-SHAPE identifies at least 10 nucleotide sequences where Gag binds to the genome. These translate into four exposed structural regions when mapped to the published 3D structure of *psi*, together with a cross linked region in the poly(A) stem loop whose 3D structure has not yet been modeled.

## The XL-SHAPE procedure

**Figure 1:**
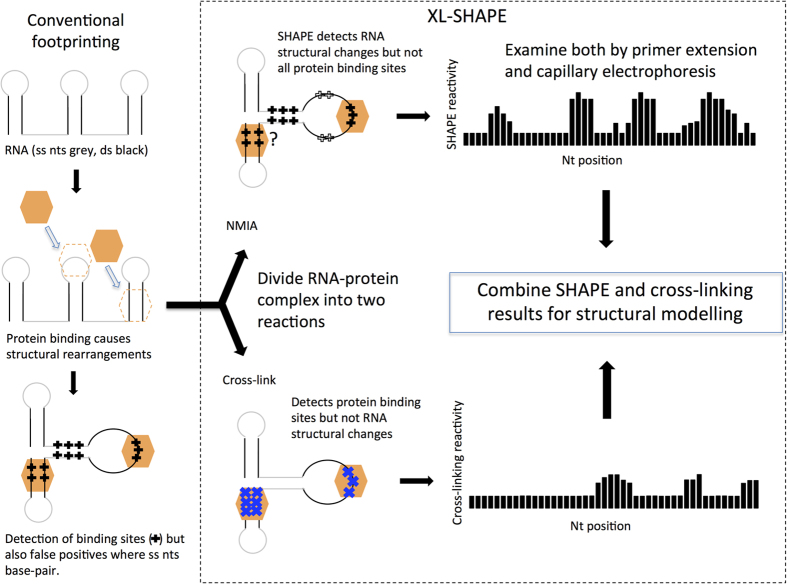
Footprinting vs XL-SHAPE. Protein binding to RNA leads to structural change. Current footprinting techniques (left hand panel) can fail to differentiate between this structural change and a protein binding site-for instance in a single stranded (ss) region that becomes base-paired, where ss probing reagents are used. Such reagents can also fail to detect protein binding in double stranded (ds) regions of the RNA. In XL-SHAPE, to improve accuracy and sensitivity of RNA-protein complex modeling, samples containing the complex are divided, with one half of the sample probed with SHAPE reagents and the other half cross-linked with UV at 254 nm. SHAPE provides a detailed picture of structural changes that occur in the backbone, rather than pinpointing the interactions with proteins—these are specifically detected by cross-linking. Acylation and cross-linking sensitivity at each nt are measured using a capillary electrophoresis platform, and the data are used together to model the RNA-protein interaction.

The pipeline for conducting XL-SHAPE is depicted in Fig. 1. SHAPE utilizes acylating reagents to modify the 2′OH of the ribose moiety where the backbone is flexible, which generally correlates with single stranded regions. As these interact with the ribose rather than the nucleobase they probe all four nucleotides. The bulky adduct blocks reverse transcription; termination products can be electrophoresed alongside sequencing ladders to quantitate the relative amounts of SHAPE adduct at each nucleotide.

Capillary sequencing using fluorophore-labeled primers is used to make the technique high-throughput^15^.

UV irradiation at 254 nm induces cross-links between RNA and protein as nucleobase excitation at this wavelength leads to the formation of a covalent bond with a nearby amino acid side-chain^16^. Almost all amino acid side chains are reactive, as are each of the nucleobases, although uracil cross-links most readily^17^,^18^,^19^. Several techniques to study RNA-interaction sites have been developed based on cross-linking, either in cells^20^, or on RNA-protein complexes extracted from them^21^. Digestion of the cross-linked RNA-protein complex leaves a peptide bound to the RNA which blocks reverse transcription; hence primer extension can be used to identify the cross-link site. Scarcity of starting material and low sensitivity has hindered the development of such a technique^16^. Here, we use a capillary method of analysis to examine both cross-link sites and NMIA acylation sites within the same RNA-protein complex, allowing rapid and accurate visualization of both protein interaction sites and structural change.

## Validation of XL-SHAPE with HIV Tat/TAR

The HIV TAR RNA and its ligand the Tat protein have been studied by many techniques including NMR. A Tat peptide (RKKRRQRRR^22^,^23^,^24^,^25^,^26^) is known to interact with TAR in a similar manner to the full length Tat protein. We first considered the sensitivity of our system. Acylation using SHAPE reagents readily occurs^27^, however photo cross-links are likely fewer. As we may be detecting a low signal we included additional controls to minimize ‘noise’. Individual fluorophores influenced the SHAPE readout and compensation was made for this (Fig. S1).

**Figure 2:**
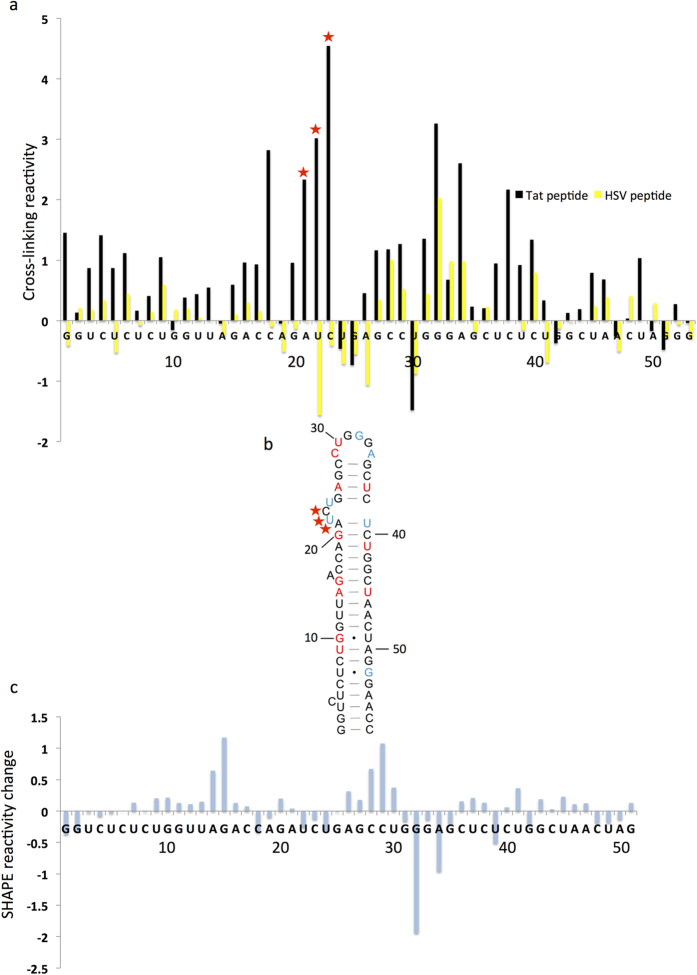
Examination of TAR RNA-Tat peptide complexes by high-throughput cross-linking or SHAPE. (**a**) TAR RNA cross-linking reactivity with Tat peptide or HSV peptide. Cross-linked or non-cross-linked RNA was incubated with/without peptide and reverse transcribed with 6FAM or VIC-labeled primers in the four ways described in Fig. S1. The extent of cross-linking was calculated as described in the text, interactions with Tat peptide are shown in black; yellow bars show HSV peptide samples. Stars indicate nucleotide positions with cross-linking reactivity statistically significantly higher in the presence of Tat peptide than HSV peptide (p < 0.05 by paired t-test), and amongst the top 20% of values for the Tat and HSV datasets. (**b**) NMIA reactivity changes upon peptide binding. TAR RNA and the TAR-Tat complex were probed with NMIA or DMSO only. NMIA reactivity changes upon Tat binding are mapped onto the secondary structural model of TAR RNA. Nucleotides are annotated colorimetrically such that blue represents a decrease in SHAPE reactivity of more than 0.2 units in the majority of repeats and red represents the equivalent increase. (**c**) Difference in NMIA reactivity before and after Tat addition, by subtraction of the TAR NMIA value from the TAR plus Tat value.

The strongest cross-links should occur where protein and RNA interact stably, however transient nonspecific interactions might generate cross-links. The Tat peptide was therefore compared to a control peptide with a high proportion of basic residues that was known to interact nonspecifically with TAR (HSV peptide, LGDPKPKKNKKPKNPC^23^). TAR RNA was renatured and incubated alone, or with either Tat or HSV peptide, and either cross-linked or not cross-linked. Non cross-linked samples controlled for differences in the reverse transcriptase termination pattern caused by stable RNA structural rearrangements upon protein binding, or the presence of residual protein. These values were subtracted from those for cross-linked samples (see equation 1, Materials and Methods) The results, applied to both Tat and HSV peptide experiments, are shown in Fig. 2a. We considered that true, specific cross-links should generate the highest peaks (top 20%), which should be statistically significantly higher than the corresponding peak in the nonspecific protein control. The sites fulfilling these criteria for the average of three independent experiments shown in Fig. 2a correspond to A21, U22 and C23 which form an arginine binding pocket to which Tat peptide has previously been shown to bind^25^,^28^. The main interaction site according to NMR studies is A21-U22, although intermolecular NOEs were also seen with C23, U24 and G25, as well as the pyrimidine-rich region on the opposite side of the helix which includes U37 and C38^28^. Residues in the three base-pairs below the bulge have also been shown to be important for Tat binding^24^. Interestingly the cross-linking data are statistically significantly higher at U37, although this nucleotide was just outside the top 20% of values, and C38, which is paired with G25, although displaying high cross-linking reactivity on average, was not statistically significant from the HSV control over three experiments. Other nucleotides such as G1 and C4 are not known to interact with Tat peptide, but displayed relatively high cross-linking reactivity, although not significantly different from the HSV peptide. We therefore considered the inter-experimental variability and how many replicates it is necessary to perform. Figure S2a shows the difference in cross-linking reactivity between Tat and HSV peptides at each nucleotide, for three independent experiments. When we included the data from an additional experiment, shown in Fig. S2b, and the average in S2c, the cross-linking becomes visible across the Tat binding pocket from G20-C23, as well as at C38. Thus, as can be seen from the variation between replicates in Fig. S2a and S2b, inter-experimental variation is relatively high and it is necessary to perform multiple replicates. Although with three replicates the main, high-affinity interaction site is identified, the periphery of the binding site becomes visible with further experiments. Such inter-experimental variation is a limitation of primer extension assays analysed by capillary sequencing and has been observed by other groups when looking at RNA-RNA or RNA-protein interactions^29^. We recommend therefore analysing a minimum of four independent experiments, if the protein of interest is likely to make multiple contacts with the RNA.

TAR RNA and TAR-Tat peptide samples were also probed with the SHAPE reagent NMIA (N-methylisatoic anhydride). To ascertain SHAPE reactivity before addition of Tat peptide, RNA or RNA-Tat peptide were probed with NMIA in DMSO or with DMSO only. Reproducible SHAPE reactivity changes were considered to be a difference of more than 0.2 units that was consistently seen in the majority (>66%) of experiments. These are marked colorimetrically on Fig. 2b, and the differences in SHAPE reactivity are plotted in Fig. 2c. Interestingly, the largest reactivity change is at the apical loop, where there is a consistent decrease in reactivity upon Tat binding. Without previous three-dimensional knowledge of the interaction, or cross-linking data, it might be assumed that this decrease in SHAPE reactivity was caused by protein binding. However, the Tat peptide does not interact with this region^28^, hence the SHAPE reactivity change must result from a change in backbone flexibility in the loop. SHAPE reactivity is mainly lowered within the arginine-binding pocket, presumably through peptide binding leading to a more stable backbone, although it increases at U37. Tat peptide binding has been shown to promote formation of the closing pair of the TAR bulge at A21-U39^28^, which forms only transiently when protein is not present. Within the helices above and below the arginine-binding pocket the reactivity increases slightly. Overall, our NMIA data show the changes in backbone flexibility, but crucially, would not allow prediction of protein binding sites. When NMIA data are viewed alongside the cross-linking data, a more complete picture of both binding sites and structural change is obtained.

## Calibration using Bacteriophage MS2 RNA-protein interactions

We chose the bacteriophage MS2 coat-binding protein (CBP)—stem-loop (SL) interaction to ensure that our system was sensitive enough to be generally applicable to RNA-protein interactions including those that do not have 1:1 stoichiometry^30^. The MS2 CBP-GFP protein was bacterially expressed and purified. This was incubated at 10× molar excess with the MS2 SL RNA and cross-linked as before, with 10× BSA as a negative control. Results are shown in Fig. S3 Using our previously described system of calibration, one cross-link was detected, shown with a star on both the cross-linking reactivity profile and the secondary structural model. MS2 CBP has been shown to interact with this nucleotide and other apical loop residues, as well as with the bulged A^30^. Interestingly, the cross-linking data at this bulge are only just outside statistical significance (by t-test, p = 0.056, and it is within the top 20% of values). Again, our data suggest there is a small degree of cross-linking occurring at this site. Overall, our technique identifies the MS2 binding site accurately, and does not show cross-linking elsewhere on the structure or with the nonspecific control protein BSA.

## Identification of Gag binding sites and corresponding RNA structural change within the dimeric HIV-1 leader RNA

**Figure 3:**
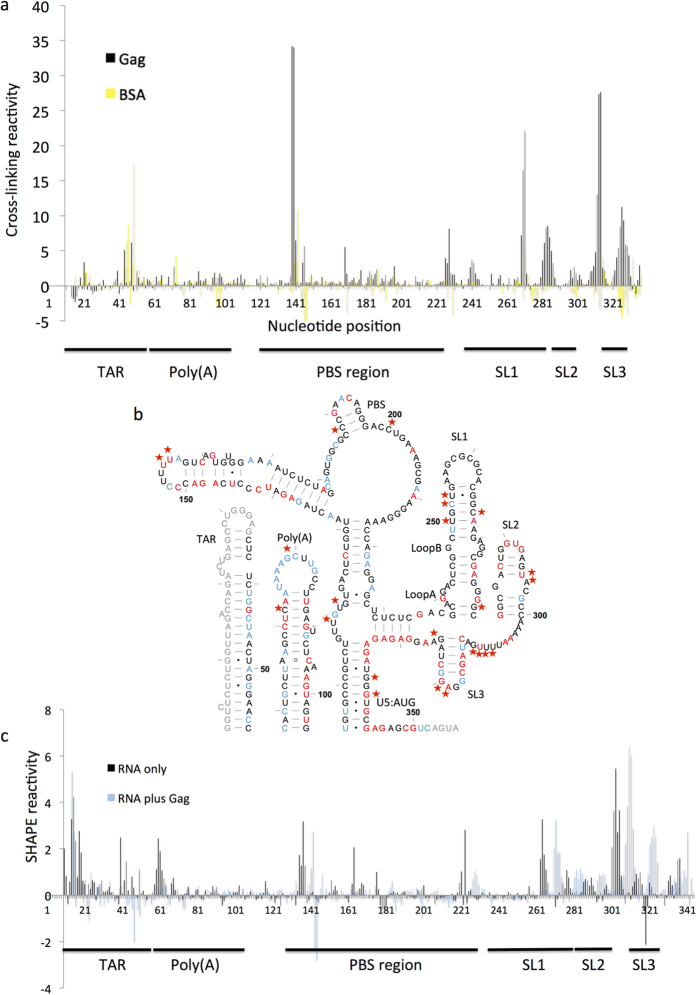
Analysis of Gag binding sites and RNA structural change on the HIV-1 RNA leader. (**a**) Cross-linking reactivity profile of the entire RNA leader, showing nucleotide number along the x-axis. Reactivities were calculated as in previous experiments. Black: reactivity profile of Gag. Yellow: reactivity profile of BSA. (**b**) NMIA reactivity changes upon Gag binding. The secondary structure of the dimer^13^ is annotated as for Fig. 2, with nucleotides for which no data were obtained in grey. Red stars indicate protein cross-linking sites that are significantly different from BSA control. Nucleotides are numbered every 50 bases with tick marks every 10. (**c**) NMIA reactivity profile for the entire leader RNA, with (blue) and without (black) Gag.

Dimerization of the HIV-1 genomic RNA is an important step in the viral lifecycle^31^. It has previously been shown that there is a structural switch in the RNA between monomeric and dimeric forms, whereby the monomer contains a pseudoknot between the dimerization initiation site (DIS) and U5 regions and the dimer contains a U5:AUG duplex, leaving the DIS exposed for dimerization at the tip of SL1^12^,^13^. Previously, we used conditions that stabilized the structure into a dimer, and examined it by in-gel SHAPE^13^. Here we used the same renaturation conditions and RNA was examined in the presence of binding buffer, to verify its dimeric status immediately before addition of Gag protein (Fig. S4). We chose to work with Gag Δp6 as our purification techniques enable removal of the affinity tag, and previous work^32^ has suggested that removal of the p6 domain does not influence specific binding to the RNA. HIV-1 RNA was renatured as described and incubated with Gag for 15 min before cross-linking or NMIA treatment, followed by purification of the RNA, reverse transcription and analysis as for the previous experiments. Complexes were found to be at equilibrium after 15 min as longer incubation times produced similar electrophoretic mobility shifts on native polyacrylamide gels (data not shown). As multiple binding sites were expected, six experimental replicates were performed and the average is presented. Cross-linking data are shown for the entire leader in Fig. 3a, and are broken down into individual regions in Fig. S5, with numerical data presented in Table S1.

All 20 amino acid side chains have been shown to cross-link, as has each of the bases^17^,^18^. However, there is a hierarchy of cross-linking efficiency: although most amino acids were represented in a recent study of 124 distinct RNA binding proteins, no RNA was found cross-linked to glutamine, glutamic acid, asparagine or aspartic acid^33^. The same study also found that cysteine may be able to form additional cross-links in the presence of DTT, which is present in our assay. Hence our study may be able to detect cross-links at the cysteine-rich zinc knuckle domain of NC more efficiently than interactions with other regions of Gag. Of the nucleobases, pyrimidines, and particularly uracil cross-link most readily^17^,^18^,^19^ hence although we observe all four bases cross-linking to Gag it is likely that our system is more sensitive to interactions with pyrimidines than with purines. There are at least ten nucleotide sequences within the RNA that cross-link to Gag, these are also marked with stars in Fig. 3b. Figure 3b,c also show the SHAPE reactivity changes upon Gag binding (with the numerical datasets in Table S2). The 3′end of the structure contains multiple short sequences of cross-linking reactivity (Fig. S5d). Although only one or two nucleotides within each of these are statistically significantly different from the BSA control, the clustering of higher reactivity makes it likely that these whole areas represent binding sites. In fact, many of the reactive nucleotides in the middle of these clusters that don’t meet the inclusion criteria (hence are not starred) are purines and hence less reliably reactive to cross-linking.

**Figure 4:**
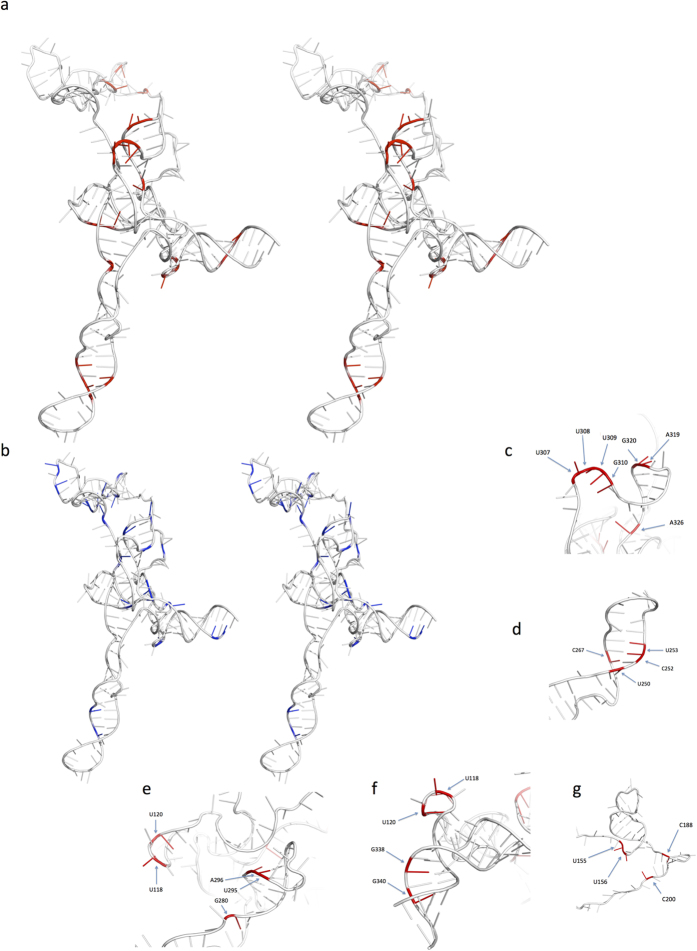
Superimposition of either XL-SHAPE derived sites indicating protein binding or SHAPE sites indicating lowered reactivity on 3D structure of HIV- 1 leader RNA nts 104–344 (a,b). Stereo (cross-eye) view of psi comparing cross-links in red (**a**) to nucleotides with lowered SHAPE reactivity in blue (**b**). The view shows SL1 at the lower extremity, the U5/AUG helix to the right. SL2 and SL3 are central; the PBS extends behind the plane of the image. (**c**) Gag binding sites in the SL3 and AU rich loop showing close proximity of protein bound bases. (**d**) Gag binding sites in SL1 proximal to the palindromic DIS sequence. (**e**) Cluster of Gag binding bases in SL2, K-turn motif and the apex of the U5/AUG helix. (**f**) Cross-linked nucleotides on the 3′ face of the U5/AUG helix showing potential Gag binding ‘face’, possibly including U118 and U120. (**g**) Gag cross-linked bases in the PBS region.

The nucleotide regions cross-linking Gag were mapped on to nucleotides 104–344 in the 3D structure of this RNA (Fig. 4a) and compared to the distribution of lowered SHAPE reactivities (Fig. 4b). Strikingly the cross-linked sites correspond to four major domains of the 3D structure of the *psi* region, which are distinct and separated in space (the poly(A) stem loop has not been modeled in 3D to date). All but a few cross-link sites are on exposed faces of the structure. Those which are not exposed are likely revealed during the conformational change occurring during Gag binding, a process which has most clearly been demonstrated for SL3^34^.

As expected, Gag cross-links with SL3, which is recognized as a major packaging signal, at A319 and G320 in the GGAG loop. 5′ of SL3 is a short AU rich sequence in which U307, 308 and 309 and G310 also cross-link. In the published 3D structure these two loops are closely adjacent and provide a binding face for Gag interaction (Fig. 4c). SHAPE does not identify any of these residues but does generate a signal flanking the SL3 bases at G317 and 321. A326 is positioned as a cross-link site where it would be accessible to Gag as SL3 unwinds.

In SL1 bases U250, C252 and U253 and C267 on opposite sides of the terminal helix present a cross-linking motif (Fig. 4d); SHAPE identifies C252 within this region. A second domain proximal to this occurs in a ‘pocket’ flanked by the proximal end of SL1 and the predicted kink turn motif. G280, U295 and A296 surround this (Fig. 4e). U118 and 120 at the apex of the U5/AUG helix also cross-link and may be part of this Gag binding face (Fig. 4e) but they may also be a domain of the U5/AUG binding site (see below). Intriguingly these two SL1 related domains flank the region recently suggested to be a critical high affinity Gag binding site^35^. Interaction of monomeric or dimeric Gag here would fit with this model.

Not previously recorded is a Gag binding site in the U5:AUG duplex on the 3′ face, G338 and 340 (Fig. 4f). SHAPE reactivity implies protein binding on the 5′ face at U105 and U107. Cross-links at U118 and U120 and a SHAPE signal at G119 may be associated with this novel Gag interacting region.

A variety of cross-linking sites and SHAPE reactivity changes are noted in the PBS (primer binding site) region. Interestingly the four bases identified by crosslinking are adjacent in 3D space (Fig. 4g) whereas SHAPE reactivity changes occur adjacent to this but are also more widely spread throughout the PBS. Interpretation of this area of the structure is problematic however as the PBS has a striking variety of structural representations in different publications with three possible structures of the upper loops and six possible variants of the lower region (reviewed in^36^) and this area is complicated by the interaction with the primer tRNA. It is likely that the PBS can adopt several conformations at various stages in the viral lifecycle. Thus we would classify the PBS as a probable Gag binding site.

There are several central ‘embedded’ bases A133, G221, A222 and A225 and G298 whose SHAPE reactivity decreases but at which no cross-linking is seen. Indeed comparing the overall reactivity of many structural motifs in the Gag bound model suggests that certain motifs, such as SL3, are destabilized by Gag binding, whereas others, including the 3-helix junction above the U5:AUG duplex and the apical loop of the poly(A) stem are stabilized by Gag binding. These bidirectional changes are consistent with the structural switch described below.

## Discussion

Previous studies that featured cross-linking of unmodified RNA to proteins have identified a minimal number of binding sites^16^,^21^,^37^. By applying capillary sequencing technology with appropriate controls, and combining this with a thorough secondary structural analysis, we show that it is possible to use XL-SHAPE to scan a large RNA, identifying multiple areas of RNA-protein interaction and concurrent structural change. Uniquely, XL-SHAPE distinguishes between specific protein interaction sites and changes in RNA secondary structure away from these sites. XL-SHAPE revealed four major Gag interacting faces of the *psi* structure and also the poly(A) stem loop. Amongst these are sites in common with a previous footprinting study using Gag-GST^14^ that identified similar regions within the PBS loop, loop B of SL1 and the SL3 loop as well as nucleotides at the base of SL3. Enhanced single-stranded cleavage and reduced double-stranded cleavage was previously seen in SL2 but might have been a knock-on effect on RNA structure or a Gag binding site^14^. XL-SHAPE detects a similarly increased flexibility of nucleotides in both the SL2 stem and loop, and also cross-linking to its 3′ stem. This latter now connects in 3D space to Gag interaction sites on SL1. In the previous study the 5′ side of the U5-DIS pseudoknot/U5:AUG interaction appeared to bind Gag. This study was performed on 90–95% monomeric RNA, hence the RNA is likely to have been in the pseudoknot form. It will be interesting to determine whether this pseudoknot is reactive to Gag binding by XL-SHAPE. Interestingly, Damgaard *et al.* detected structural change/binding to loop B of SL1, as do we^14^. This suggests that the loop B binding site may function in both monomeric and dimeric RNA conformers, which is at odds with a recent study^35^. That study used full-length rather than GagΔp6 and suggested that the differences in specificity of binding between theirs and previously published work was due to their use of full-length protein. However, they did not include a comparative study with Δp6 protein; our results, showing similar binding sites on SL1, suggest that the specificity of binding to SL1, *in vitro* at least, is not affected by the p6 domain.

A previous NMR study of the interaction of SL3 RNA with NC showed interaction of NC with the 3′GAG in the terminal GGAG tetraloop^38^. Although XL-SHAPE detects interactions with the 3′AG of this GGAG moiety, it does not detect cross-linking at the second G. This could be due to the reduced cross-linking potential of guanosine, or to differences between interactions of the NC domain and the Gag protein.

XL-SHAPE detected previously unidentified Gag binding sites: the poly(A) loop and upper stem, the 3′ side of the U5:AUG helix, the GU-rich region around nt 120 and loop A of SL1. These may all reflect binding sites unique to the dimer that the virus may use to facilitate its selection over the monomer: the conformations of the U5:AUG helix and the GU-rich region change greatly during the structural switch^12^,^13^, whereas those of the poly(A) loop and loop A of SL1 display different SHAPE reactivity between monomer and dimer. It is possible that the poly(A)loop makes intermolecular contacts in the dimer that constitute a Gag binding site, or that an intramolecular interaction prevents it from binding Gag in the monomer. Until a 3D structure including the poly(A) region is solved this remains speculative. However such pseudoknots have been suggested to form in the monomer^39^. Stabilization of some of the helices was also seen on Gag binding supporting the protein interaction as favouring a particular RNA conformation for incorporation into the budding virus. The bidirectional changes in SHAPE reactivity are consistent with a model in which Gag binds preferentially to a dimeric RNA and in doing so can melt local structures. Formation of the nucleoprotein complex leads to a stabilization of the RNA dimer. This model has attractive resonances with the concept of the change from ‘loose’ to ‘tight’ dimer by the viral RNA as it is encapsidated^40^. XL-SHAPE also illustrates that acylation sensitivity within a protein binding site can increase, decrease or remain similar, showing that SHAPE reagents alone are not sensitive footprinting reagents. XL-SHAPE, however, alongside other recently published powerful techniques^19^,^41^ now gives us the opportunity to solve RNA-protein interactions and their dynamic structural changes at single nucleotide resolution.

## Online Methods

### RNA constructs

RNA was prepared by *in vitro* transcription, using templates with T7 promoters. Templates were prepared by PCR using 1 × Biomix red (Bioline) and 0.5 μg template DNA. Templates used were pSVC21^42^ for TAR and HIV-1 leader RNA. For MS2 RNA, a mixture of two oligonucleotides (MS23, 5′GGGGGGGGGGAGATGGGTAATCCTCATCTTTACTAGAGTCGACCT GCAGACATGGGTAATCCTCATGT3′, and MS25, 5′ACATGAGGATTACCCATGTCTGCAGGTCGACTCTAGTAAAGATGAGGATTACCCATCTCCCCCCCCCC3′) was mixed, heated to 95 °C and slow-cooled to 4 °C. Primers used were: TAR forward, 5′TAATACGACTCACTATAGGCCTTCGGGCCAAGGTCTCTCTGGTTAGACC3′; TAR reverse, 5′CACTACTTGAAGCACTCAAGG3′, MS2 Forward, 5′TAATACGACTCACTATAGGGGCCTTCGGGCCAAACATGAGGATTACCCATGTCTGCAGGTCG3′ MS2 reverse, 5′GAACCGGACCGAAGCCCGATTTGGATCCGGCGAACCGGATCCTGGGGGGGGGGAGATGGG3′, HIV forward, 5′TAATACGACTCACTATAGGGTCTCTCTGGTTAGACCAGATCTG; HIV reverse, CTTTCCCCCTGGCCTTAACC3′. PCR products were purified on 1% agarose gels in Tris-borate ethylenediamine tetra-acetic acid (TBE) (89 mM Tris base, 89 mM boric acid, 2 mM ethylenediaminetetraacetic acid (EDTA)). Gels were stained with 1.3 μM ethidium bromide to visualize templates, which were then purified by gel extraction (QIAgen). *In vitro* transcriptions were performed with the Ambion T7 Megascript kit and purified with Ambion MegaClear columns. Briefly, each transcription reaction contained 7.5 mM deoxyribonucleotide triphosphates (dNTPs), 1 × reaction buffer, 2 μg of template DNA and 2 μl of T7 RNA polymerase and was incubated for 3 h at 37 °C. DNA was degraded with 4 U of DNase (TURBODNase, Applied Biosystems) for 1 hr at 37 °C. RNA was eluted from MEGAclear columns (Applied Biosystems) in water and stored at −20 °C. The structural integrity of each RNA was assessed on denaturing polyacrylamide gels (5% polyacrylamide, 7M urea in TBE).

### Protein expression and purification

Tat and HSV peptides were purchased from Thermo Scientific and resuspended in water. The pMS2-GFP construct was obtained from Addgene. MS2-GFP was amplified using primers: 5′CCCGGGATCCATGGCTTCTAACTTTACTCAG3′ and 5′GCCGCTCGAGTTATTTGTATAGTTCATCCATGCC3′, cloned into pGEX-6P-1 using *Bam*HI and *Xho*I sites, and sequenced. Gag Δp6^34^ and MS2-GFP were expressed in *E.coli* Bl21(DE3) cells. Cells were induced at an OD of 0.6, with 0.5 mM IPTG for 90 min at 30 °C. Cells were harvested and resuspended in a minimal volume of resuspension buffer (50 mM tris-HCl pH 7.5, 300 mM NaCl, 1 mM dithiothreitol (DTT), protease inhibitor (Roche Complete mini EDTA-free) 1 tablet per 10 mL) before treatment with 0.5 mg/mL lysozyme for 5 min at 4 °C. Cells were sonicated for 3 × 1 min at 50% power output in a cup-horn sonicator, with cooling, and treated with 0.5% triton X-100 for 5 min. The suspension was clarified at 12,000 × g for 20 mins; supernatant was applied to a GSTrap FF column (GE Healthcare) that had been previously equilibrated in binding buffer (PBS (140 mM NaCl, 2.7 mM KCl, 10 mM Na_2_HPO4, 1.8 mM K_2_HPO_4_, pH 7.3), 1 mM DTT) using an AKTApurifier (GE). The column was washed with binding buffer until the UV trace reached baseline and equilibrated with Prescission cleavage buffer (50 mM Tris-Cl, 150 mM NaCl, 1 mM EDTA, 1 mM DTT, pH 7). Prescission protease (660 U/5 mL column) was applied to the column and incubated for 3 h at 4 °C. A further GSTrap FF column was equilibrated in Prescission cleavage buffer and attached below the first column, to remove cleaved GST. Protein was eluted in a mixture of PBS-DTT and Prescission cleavage buffer (protein elution buffer, 142 mM NaCl, 12.5 mM Tris-HCl, 0.25 mM EDTA, 675 μM KCl, 2.5 mM Na_2_HPO_4_, 450 μM K_2_HPO_4_, 1 mM DTT) concentrated (Amicon Ultra centrifugal filter), and assessed by polyacrylamide gel electrophoresis, Western blot and Bradford assay.

### Cross-linking

The cross-linking protocol was developed from a previous study^21^. For MS2 and TAR RNA, 10.5 pmol RNA in 52.5 μl refolding buffer (10 mM Tris pH 8, 100 mM KCl, 100 μM EDTA) was heated to 95 °C for 2 min and snap-cooled on ice. For HIV-1 RNA, 10.5 pmoles per sample was prepared as 10 μM stock in H_2_0, heated at 95 °C for 2 min and snap-cooled on ice. The sample was then diluted to 1 μM in 10 mM Tris-HCl, 140 mM KCl, 10 M NaCl, 1 mM MgCl_2_ and incubated for 1 hour at 37 °C. RNA was placed at room temperature and the volume was increased to 150 μl per sample with 30 μl binding buffer (500 mM Tris pH 8, 200 mM KCl, 50 mM DTT, 1% triton X-100, 1 mM ZnCl_2_), along with 15 μl RNasin and 100× excess (w/w) yeast tRNA. Each sample was divided into two: 75 μl aliquots were added into each of two wells of a round-bottom 96-well plate. For RNA-only samples, 75 μl of Protein elution buffer was added; for RNA-protein samples, 75 μl of protein in protein elution buffer was added. Plates were incubated at room temperature for 15 min. Samples were cross-linked on ice for 2 min at default power in an XL-1500 Spectrolinker UV cross-linker (Spectronics Corporations). Non-cross-linked control samples were incubated on ice for 2 min with no cross-linking. SDS was added to 0.5% final concentration, alongside 200 μg/mL Proteinase K. Plates were incubated on heat blocks set to 55 °C for 60 min. RNA was recovered by transferring sample to 1.5 mL microfuge tubes, phenol-chloroform extraction and ethanol precipitation.

### SHAPE

RNA was renatured and RNA only or RNA-protein complexes were prepared and incubated on 96-well plates as described above. 10 mM (final concentration) NMIA in DMSO or an equal volume of DMSO only was added. Plates were incubated at 37 °C for 45 min and RNA was recovered with SDS and Proteinase K treatment and phenol-chloroform extraction as described above.

### Reverse transcription and sequencing

RNA samples from either NMIA modification or cross-linking experiments were resuspended in 12 μl 2.1 mM Tris pH 8.0, 42 μM EDTA, with 5 nmol primer (designed to bind to the 3′end of the RNA and labeled with either 6FAM or VIC). For SHAPE probing experiments, this was always 6FAM for NMIA probed RNA and VIC for the DMSO only control. For cross-linking experiments, samples for the fluorophore controls were labeled with either 6FAM or VIC. For cross-linked and non cross-linked experiments on RNA-protein complexes, the RNA-protein sample was reverse transcribed with 6FAM and the RNA only control with VIC. Primer annealing took place at 85 °C for 1 min, 60 °C for 5 min and 35 °C for 5 min. 8 μl extension mix (100 U Superscript III reverse transcriptase, Invitrogen, 1.5× RT buffer (Invitrogen), 12.5 mM DTT, 1.25 mM dCTP, dATP, dUTP and 7-deaza dGTP was added and reverse transcription was allowed to proceed at 55 °C for 1 h. Each pair of samples to be analyzed together was combined and RNA was hydrolyzed at 95 °C for 2 min with 200 mM NaOH, followed by 5 min at 4 °C and the addition of an equal amount of HCl and the sequencing ladders. These were prepared from the same DNA templates used for *in vitro* transcription, using the Jena DNA cycle sequencing kit and 1.3 nmol primers labeled with either NED or PET. Each sequencing reaction contained 50 ng template, 1× sequencing kit buffer, enzyme and extension mix as per the manufacturer’s instructions. Cycle sequencing was for 5 min at 95 °C, then 30 cycles at 95 °C for 30 s, 37 °C for 30 s and 70 °C for 1 min (Labnet Multigene thermocycler). The amount of ladders and RT products combined to be sequenced was titrated to ensure that signals from the fluorophores did not bleed into one another. Combined cDNA samples and ladders were then purified by ethanol precipitation, resuspended in 10 μl and dried onto sequencing plates before being resuspended in 30 μl formamide and fractionated by capillary electrophoresis (Applied Biosystems 3730xl analyzer).

### Correction for fluorophore

This system, analyzed with SHAPEfinder software^43^, uses fluorophore-labeled cDNAs (6FAM for the experimental sample and VIC for the control, the software calculates how much larger the 6FAM peak is relative to the matched VIC control peak). To ensure that the use of different fluorophores was not influencing the results, cross-linked TAR RNA was reverse transcribed using either a 6FAM or a VIC labeled primer. cDNAs were analyzed by capillary electrophoresis alongside sequencing ladders (Fig. S1A). Surprisingly inclusion of one or other fluorophore led to slightly different termination patterns. These patterns were reproducible and were also present, but different, when non cross-linked RNA was reverse transcribed with either 6FAM or VIC-labeled primers (S1C). As the only difference between these experiments was the primer fluorophore, this presumably affects the stability of the RNA-cDNA complex or the processivity of the enzyme. However, these differences were smaller than those occurring upon addition of Tat peptide (Fig. S1B and D). Therefore, background differences attributable to the fluorophores were subtracted by including fluorophore control samples that measured the differences in RT termination between 6FAM and VIC labeled primers for both cross-linked (Fx control, see Fig. S1A for an example) and non cross-linked (Fnox control, example shown in Fig. S1C) RNA. At least three repeats of each control were performed and the average at each nucleotide position was subtracted from the corresponding experimental results. These were performed as follows: For cross-linked samples, the 6FAM-labelled cDNA originated from the cross-linked RNA-protein complex and the VIC-labeled cDNA was made from the cross-linked RNA without protein (example shown in Fig. S1B). For the non cross-linked samples, the 6FAM-labelled cDNA came from the non cross-linked RNA-protein and the VIC labeled cDNA came from the non cross-linked RNA with no protein (example shown in Fig. S1D). SHAPE analysis involves normalizing the results of each capillary sample relative to themselves^43^, however as we were comparing cross-linked and non-cross-linked RNA samples with and without protein and were expecting larger differences amongst our cross-linked/protein-containing samples, we normalized all samples for each experiment using the average normalization value for our Fx controls. Thereafter, we calculated the amount of RT termination at each nucleotide that could be attributed to RNA-protein cross-links by the following equation (equation 1):
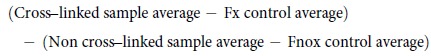
Using the example given in Fig. S1, this would be (b–a)–(d–c).

### Analysis

Traces were aligned with sequencing ladders and integrated using SHAPEfinder software. Mobility shift controls were first performed for each primer as described^43^. For NMIA probing experiments, areas of the negative control peaks were subtracted from the NMIA-probed sample peaks. Normalization of the data was performed as described^43^, by dividing the value of the peak at each nucleotide by an average of the highest 8% of results, not including outliers, defined to be above the 3^rd^ quartile plus 1.5 times the interquartile range. For NMIA experiments, each sample was normalized in this manner. For cross-linking experiments, Fx control samples were normalized in this manner and their average value used to normalize the rest of the experimental samples. In order to generate a value of cross-linking magnitude, the value of the 6FAM minus VIC peak at each nucleotide position for the fluorophore control cross-linked sample was subtracted from its counterpart in the experimental cross-linked sample, and the value of the fluorophore control for the non cross-linked sample was subtracted from its counterpart in the non cross linked control sample. The non-cross-linked data were then subtracted from the cross-linked data at each nucleotide position, to give the amount of signal attributable to RNA-protein cross-links.

## Additional Information

**How to cite this article**: Kenyon, J. C. *et al.* A novel combined RNA-protein interaction analysis distinguishes HIV-1 Gag protein binding sites from structural change in the viral RNA leader. *Sci. Rep.* **5**, 14369; doi: 10.1038/srep14369 (2015).

## Supplementary Material

Supplementary Information
