# Supplementary material for: A novel combined RNA-protein interaction analysis distinguishes HIV-1 Gag protein binding sites from structural change in the viral RNA leader
Source: Sci Rep. 2015 Oct 9;5:14369. doi: 10.1038/srep14369 (PMC4598872; doi:10.1038/srep14369)
Supplement: Supplementary Information [file srep14369-s1.pdf]

A novel combined RNA-protein interaction analysis distinguishes HIV-1 Gag protein binding sites from structural change in the viral RNA leader.

Julia C. Kenyon, Liam J. Prestwood and Andrew M.L. Lever

Fig S1

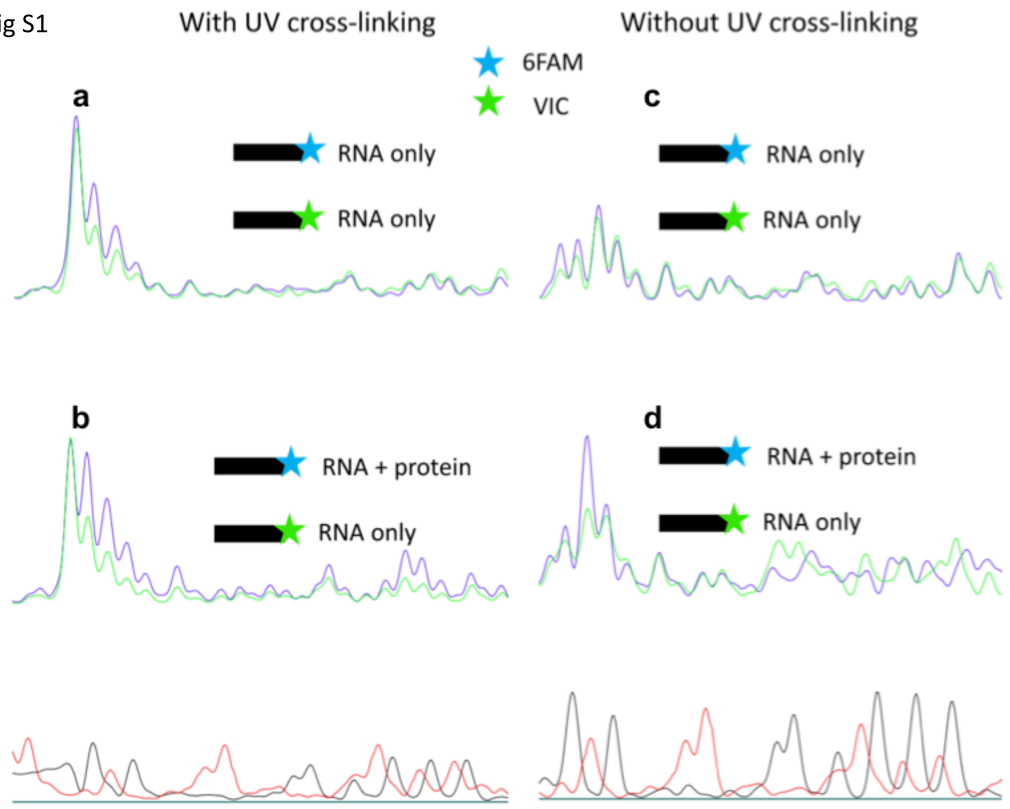

Figure S1

The experimental set-up to calculate specific cross-linking

TAR RNA or RNA-protein complexes were incubated with/without UV cross-linking, purified, reverse transcribed using 6FAM or VIC labeled primers, and analysed. Red/black traces are sequencing ladders used to align samples.

a) Example trace for Fx control samples. RNA was cross-linked and reverse transcribed with either 6FAM or VIC labeled primer, shown in blue and green respectively. b) Experimental cross-linked RNA-protein sample. RNA only was reverse transcribed with a VIC labeled primer, RNA-protein with a 6FAM labeled primer. c) Fnox control sample. Non cross-linked RNA was reverse transcribed with either 6FAM or VIC labeled primer. D) Non cross-linked sample. RNA was incubated with/without protein; the RNA only was reverse transcribed with VIC labeled primer, the RNA-protein was reverse transcribed with 6FAM labeled primer.

Fig S2a

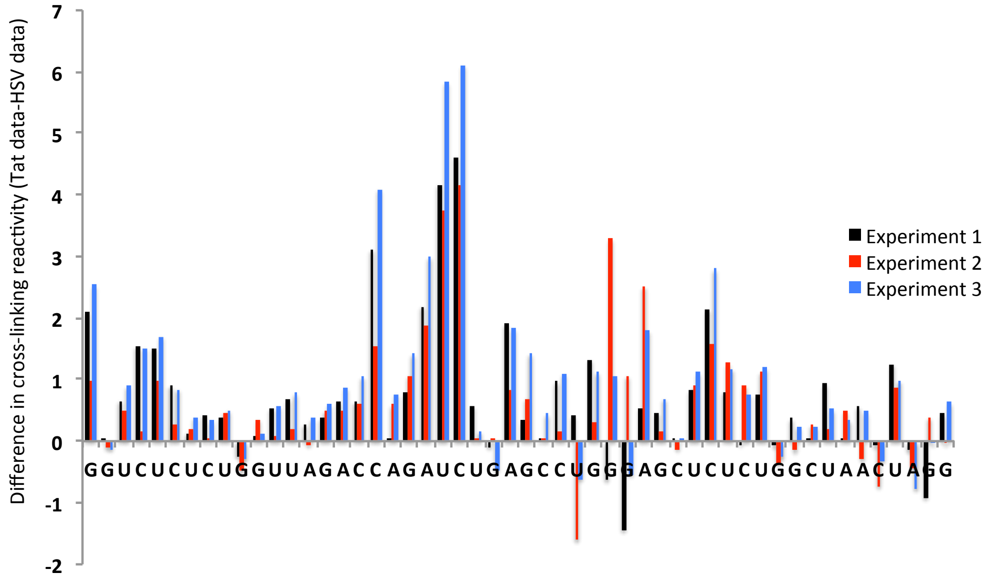

Figure S2b

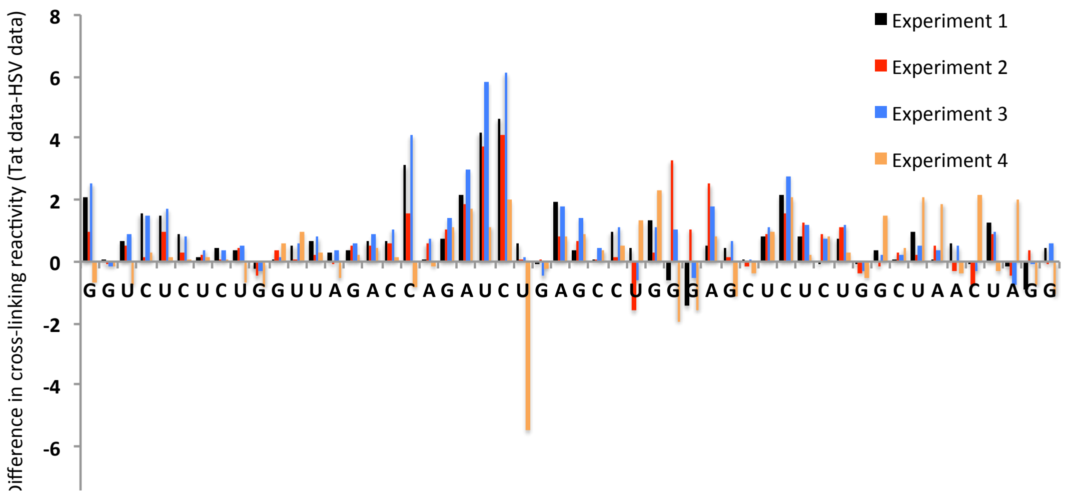

Figure S2c

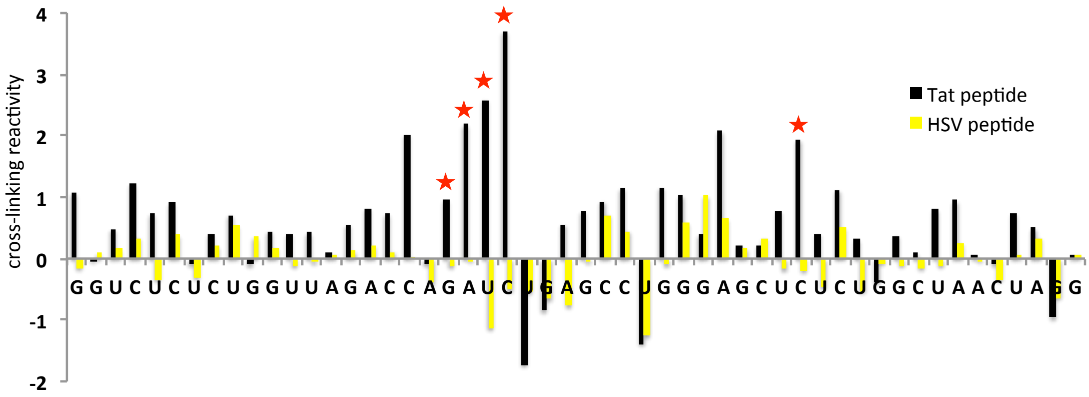

Figure S2
Inter-experimental variation analysis
a)Cross-linking sensitivities for Tat peptide broken down into individual experiments. Data are presented as Tat peptide cross-linking minus HSV control peptide cross-linking at each nucleotide position. b) Cross-linking sensitivities for individual Tat peptide experiments as 2a, with the inclusion of a fourth experiment. c) Average cross-linking reactivities for Tat peptide and HSV peptide, taking into account four independent experiments. Stars represent those nucleotides showing reactivity in the top 20% of values and statistically significantly different from the HSV control.

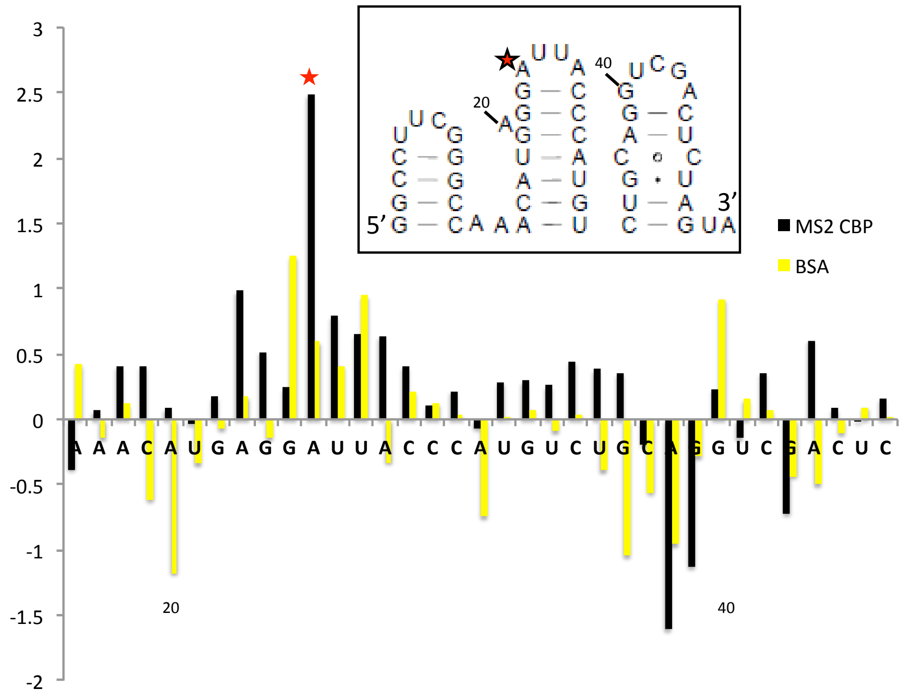

Figure S3

Validation of the cross-linking experimental set-up using MS2 RNA and Coat binding protein.

Cross-linking reactivity profile of the interaction of MS2 CBP, shown in black, or BSA, shown in yellow, with the MS2 RNA construct. Cross-linking reactivities were calculated as above, and those nucleotides where reactivity is statistically significantly higher than in the BSA control ($p<0.05$, paired t-test) are shown with stars. Inset: secondary structural model of the RNA, containing MS2 stem-loop (centre) and nonspecific stem-loops (5' and 3'). Stars indicate the position of statistically significant cross-link sites as above. Data were not obtained for the small stem-loop at the 5' of the RNA. Nucleotide position (each 20 nt) is marked.

Figure S4

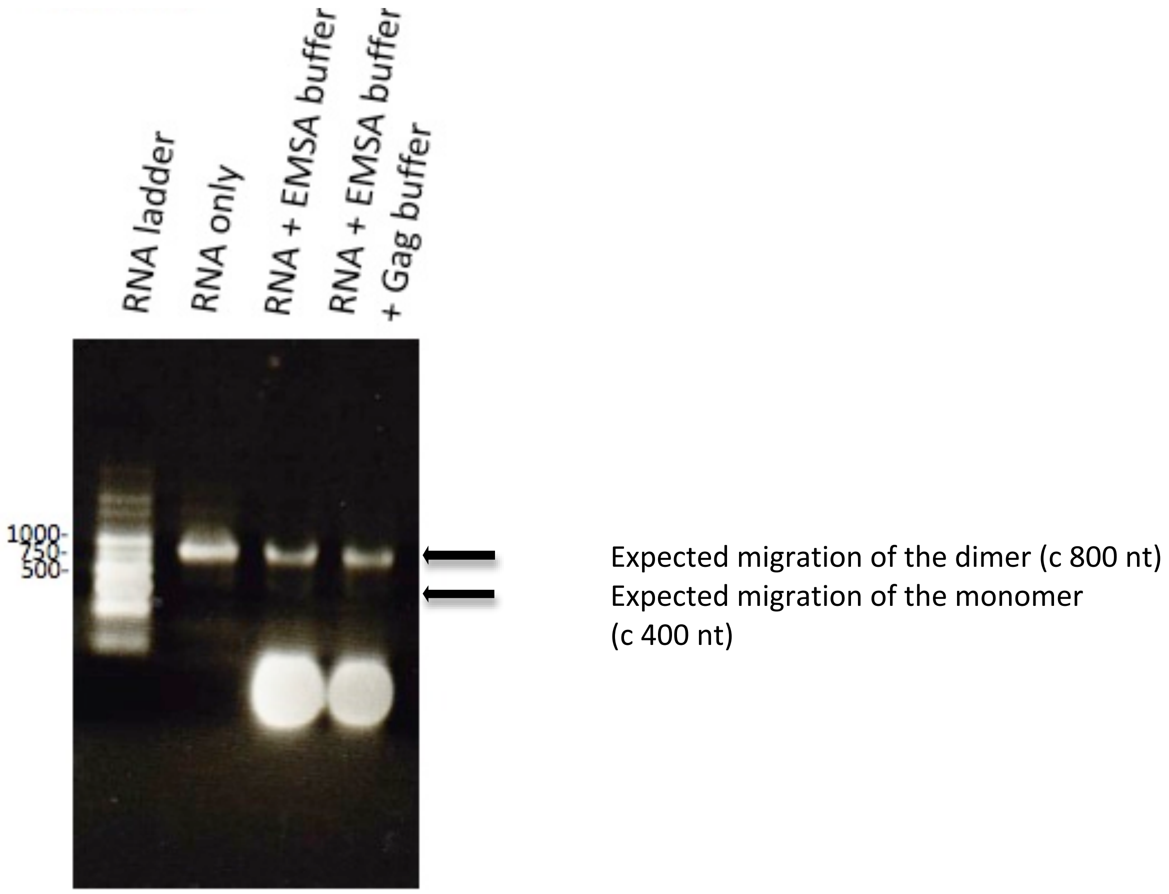

Figure S4
Agarose gel electrophoresis confirming dimeric nature of HIV RNA examined by XL-SHAPE

HIV-1 RNA was renatured in the same manner as for cross-linking experiments. EMSA buffer (binding buffer) with or without Gag buffer (protein elution buffer) was added to mimic conditions just before addition of Gag. The dimeric nature of the RNA was assessed by native agarose electrophoresis and ethidium bromide staining. The band of low molecular weight visible in lanes containing EMSA buffer is excess tRNA added to minimize nonspecific interactions between HIV RNA and protein. The distinct migration of the dimeric HIV RNA at around 800nt shows that the tRNA is not binding to the HIV RNA under these conditions.

S5a

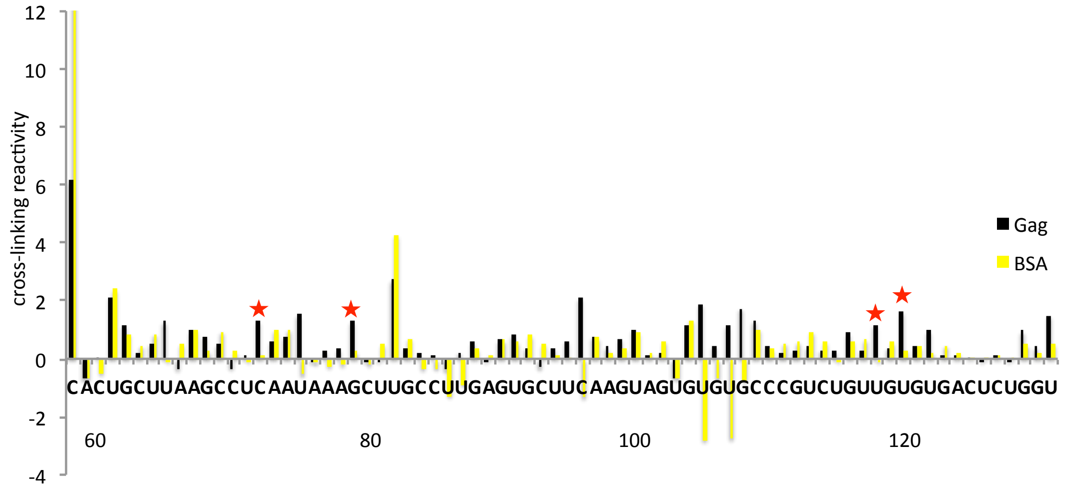

cross-linking reactivity
12
10
8
6
4
2
0
-2
-4
Gag
BSA
CACUGCUUAAGCCUCAAUAAAGCUUGCCUUGAGUGCUUCAAGUAGUGUGUGCCCGUCUGUUGUGUGACUCUGGU
60
80
100
120

S5b

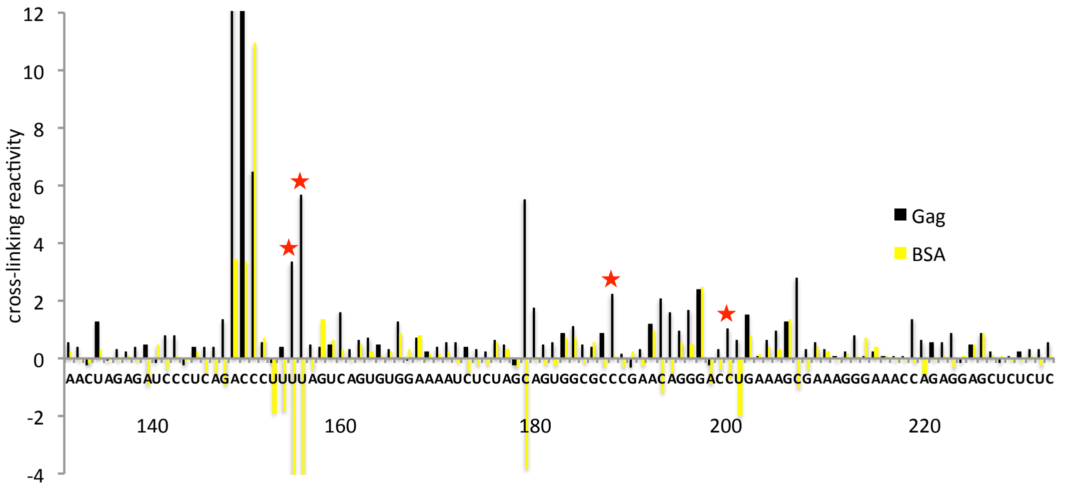

cross-linking reactivity
12
10
8
6
4
2
0
-2
-4
Gag
BSA
AACUAGAGAUCCCUCAGACCCUUUUAGUCAGUGUGGAAAAUCUCUAGCAGUGGCGCCCGAACAGGGACCUGAAAGCGAAAGGGAAACCAGAGGAGCUCUCUC
140
160
180
200
220

S5c

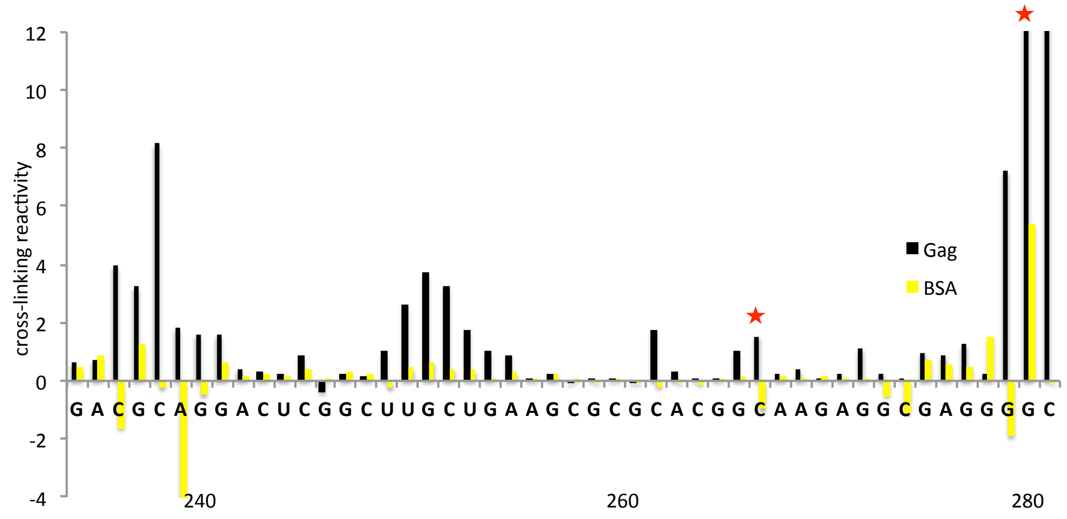

S5d

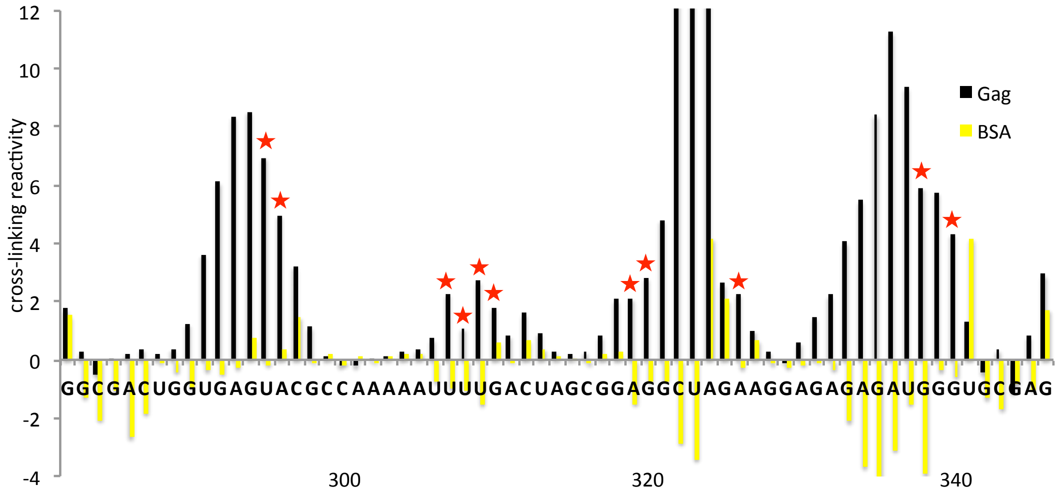

Figure S5

Expanded cross-linking reactivity profiles of sections of the RNA leader, taken from Figure 3a.

Scales have been adjusted so as to allow clearest visualization of lower peaks; refer to figure Figure 3a for the highest/lowest values. The other regions are shown approximately as: a) the Poly(A) stem-loop into the U5 region. b), the PBS region to SL1. c) SL1 and d), SL2-Gag AUG.

Table S1

NT number	Sequence	RNA-Gag XL	RNA-BSA XL	p value (t-test)
25	G	-1.823202081	1.652972087	0.136109141
26	A	-2.272064477	-0.124844905	0.229292587
27	G	-1.524161838	0.283219273	0.332264902
28	C	-0.330675358	-0.11411598	0.896361558
29	C	1.181573827	0.632229768	0.587210839
30	U	-0.43997822	2.330382041	0.247266864
31	G	3.38120363	2.183147945	0.675694202
32	G	1.8406444	0.452313383	0.339986092
33	G	-0.524952359	1.842731866	0.119163905
34	A	0.479619654	-0.604230231	0.166648278
35	G	-0.59752962	0.356414123	0.382868502
36	C	-1.072895586	0.243566738	0.39351019
37	U	-0.720483175	0.029854254	0.621594322
38	C	-0.739805816	-0.522473977	0.845123481
39	U	0.240589127	-0.143888386	0.62848821
40	C	0.482772258	-0.327510569	0.09001519
41	U	-0.608197068	0.079162806	0.580773986
42	G	0.819523877	0.478706972	0.625810613
43	G	-0.347352092	0.020686862	0.550240551
44	C	-0.037586458	-0.463938798	0.504977385
45	U	-0.15978377	-0.357290438	0.638560476
46	A	-0.261217507	0.123498932	0.664179691
47	A	-0.189360656	-0.270540278	0.886680895
48	C	0.60143374	-0.141549398	0.210282974
49	U	0.957032472	0.078177885	0.073455139
50	A	2.153357017	0.749997611	0.18906767
51	G	-1.072882017	0.655450538	0.460287511
52	G	0.418306857	0.946186974	0.680817061
53	G	-0.433613442	2.537293361	0.505030767
54	A	5.114246236	6.602658799	0.596124387
55	A	0.481070014	8.766102146	0.189285663
56	C	-0.179463339	-2.532889517	0.312466258
57	C	0.68760715	3.112047346	0.554858327
58	C	6.157289336	17.47822907	0.189833331
59	A	-0.710432034	-0.698059265	0.983703718
60	C	0.052342354	-0.505961718	0.248783056
61	U	2.150976295	2.403360573	0.89628373
62	G	1.167428555	0.87158658	0.77742387
63	C	0.218229629	0.460722672	0.661545723
64	U	0.534124267	0.866801553	0.323043529
65	U	1.29497661	-0.063464896	0.281799986
66	A	-0.353825372	0.519921685	0.095063173
67	A	0.967754591	1.001448203	0.964455383
68	G	0.796719958	0.260746142	0.449908858

69	C	0.510913116	0.941118519	0.326978581
70	C	-0.318029486	0.247818533	0.288830702
71	U	0.130384292	-0.079959024	0.614251842
72	C	1.310373123	0.144266474	0.010499474
73	A	0.606952725	0.993045344	0.632697299
74	A	0.757757734	1.005367631	0.868825493
75	U	1.535349962	-0.548452826	0.21892407
76	A	-0.105475662	-0.153304438	0.919288272
77	A	0.279272333	-0.265572005	0.064345442
78	A	0.336733531	-0.209479932	0.086169412
79	G	1.295196934	0.243851014	0.037235049
80	C	-0.038960529	-0.19190968	0.651956548
81	U	-0.046225385	0.51269457	0.467501701
82	U	2.729859339	4.271292822	0.750907795
83	G	0.38848639	0.704376594	0.681382797
84	C	0.187415484	-0.349757688	0.042070666
85	C	0.121360022	-0.324857881	0.33995877
86	U	-0.388026779	-1.276017781	0.050200388
87	U	0.230614365	-0.888728798	0.02430485
88	G	0.591065202	0.344722853	0.476418515
89	A	-0.032726349	0.158032081	0.487375258
90	G	0.644661708	0.644895435	0.999506052
91	U	0.821919081	0.593017727	0.757100646
92	G	0.39166654	0.818760418	0.07642886
93	C	-0.315662401	0.518462185	0.183655886
94	U	0.382755927	0.113715475	0.44621558
95	U	0.6263508	0.051486913	0.062198949
96	C	2.099665358	-1.316373758	0.06970933
97	A	0.771482657	0.729050966	0.918312956
98	A	0.414849191	0.221096364	0.559580934
99	G	0.69047353	0.372473429	0.50565412
100	U	1.017699096	0.905941546	0.920586565
101	A	0.131375233	0.194657768	0.838422215
102	G	0.201033813	0.610355804	0.250793382
103	U	-0.648735408	-0.702467457	0.942595181
104	G	1.161210445	1.346921523	0.958007976
105	U	1.897324027	-2.810286478	0.093665341
106	G	0.455364125	-0.700682907	0.120423771
107	U	1.159233562	-2.745004705	0.126045146
108	G	1.74477625	-0.765809121	0.144449891
109	C	1.317258024	1.023916774	0.548778558
110	C	0.456228837	0.323089783	0.663892514
111	C	0.239754307	0.548005324	0.339865915
112	G	0.302011045	0.588517163	0.393207254
113	U	0.426766084	0.933916621	0.253471551
114	C	0.320293862	0.583585932	0.218333025

115	U	0.277840358	-0.011699756	0.36120578
116	G	0.885888418	0.608574247	0.460849137
117	U	0.315964748	0.671052785	0.404256772
118	U	1.165205118	-0.086187578	0.007634514
119	G	0.398702754	0.565303428	0.444780192
120	U	1.632275832	0.304072049	0.01244675
121	G	0.431439579	0.404036675	0.915699877
122	U	0.959013452	0.200665015	0.048273141
123	G	0.098019693	0.46850339	0.419062834
124	A	0.144193775	0.165290469	0.878035716
125	C	0.067875299	0.070867827	0.9662463
126	U	-0.020753461	0.030857596	0.721964481
127	C	0.152848292	0.089758637	0.865085749
128	U	-0.019009483	0.019617036	0.922547752
129	G	0.959983336	0.485693461	0.195851169
130	G	0.416997422	0.166621051	0.385341982
131	U	1.487971149	0.5159541	0.14073296
132	A	0.544934874	0.232829986	0.263289993
133	A	0.407860971	-0.038119919	0.070710894
134	C	-0.205459827	-0.191294435	0.967359776
135	U	1.281603732	0.289514769	0.169042362
136	A	-0.060650105	-0.032059254	0.911628002
137	G	0.34837741	-0.089661125	0.340069389
138	A	0.220947281	0.113307048	0.689017858
139	G	0.370797645	-0.095053645	0.076486211
140	A	0.463196095	-0.941396377	0.325153542
141	U	-0.135035335	0.505107267	0.844983369
142	C	0.81303321	-0.385978572	0.002126069
143	C	0.773413312	0.000367252	0.015930169
144	C	-0.233355915	-0.05633389	0.39938304
145	U	0.384477898	0.209153686	0.681620231
146	C	0.398355479	-0.452287905	0.158628443
147	A	0.436583906	-0.574813228	0.088746945
148	G	1.348344531	-0.927875911	0.456573324
149	A	34.21448581	3.432985012	0.245469061
150	C	33.99361754	3.368175708	0.165015726
151	C	6.50583042	10.95823315	0.564003827
152	C	0.577192836	0.686938297	0.987870413
153	U	-0.153024679	-1.894796882	0.313985139
154	U	0.422366806	-1.839076627	0.124042798
155	U	3.337418769	-6.488752105	0.00783585
156	U	5.669132021	-6.072670405	0.011202324
157	A	0.508120459	-0.388062231	0.223380879
158	G	0.434877322	1.375883945	0.318585183
159	U	0.474655663	0.680714873	0.688575111
160	C	1.641547534	0.314831301	0.423175646

161	A	0.338314607	-0.077114913	0.223563734
162	G	0.624563195	0.480782915	0.731867991
163	U	0.715174038	0.267504048	0.317961995
164	G	0.517419977	0.120196594	0.726180234
165	U	0.294451689	0.272942171	0.989071406
166	G	1.270068883	0.900811342	0.451406799
167	G	-0.09202745	0.31014376	0.488230028
168	A	0.705360645	0.768058494	0.941826632
169	A	0.271818301	0.194176657	0.7863013
170	A	0.376666848	0.167127051	0.385365349
171	A	0.552455415	0.208508186	0.036759893
172	U	0.572362331	-0.132180444	0.002168248
173	C	0.381440547	-0.492973935	0.025777098
174	U	0.345588391	-0.241953282	0.010166713
175	C	0.253618193	-0.218889708	0.408305493
176	U	0.631523805	0.573096954	0.907518513
177	A	0.456584428	0.29997039	0.842701744
178	G	-0.228656444	-0.315743323	0.951614501
179	C	5.54962564	-3.8247326	0.385914413
180	A	1.745103719	-0.092799453	0.422515714
181	G	0.504564761	-0.218298566	0.326453142
182	U	0.565042578	-0.260099891	0.236577569
183	G	0.84852675	0.708848998	0.807308598
184	G	1.115374573	0.723493211	0.46630896
185	C	0.448097541	-0.167460401	0.02661931
186	G	0.392884062	0.598887163	0.631692204
187	C	0.912712534	-0.302692825	0.104166773
188	C	2.257146007	0.00078414	0.037267516
189	C	0.165820489	-0.314736068	0.303219611
190	G	-0.277658936	0.251990124	0.484784966
191	A	0.346313528	-0.26247238	0.277366857
192	A	1.216070797	0.993364584	0.905834311
193	C	2.091917878	-1.175447884	0.127176475
194	A	1.637437005	-0.554143266	0.083132767
195	G	0.99031804	0.575578037	0.521819576
196	G	1.706379524	0.519633234	0.381777493
197	G	2.363379393	2.494935111	0.835806116
198	A	-0.200121316	-0.876145703	0.104900877
199	C	0.317866739	-0.419215561	0.044290949
200	C	1.058773878	-0.677027409	0.04023701
201	U	0.668266111	-2.028645355	0.013511657
202	G	1.533492235	0.822843015	0.427094153
203	A	0.110931664	0.174030214	0.854268717
204	A	0.617306438	0.37963053	0.269593401
205	A	0.984032281	0.308539308	0.353722981
206	G	1.291602168	1.329414849	0.946091938

207	C	2.803307988	-1.032447482	0.062614258
208	G	0.309937244	-0.399920026	0.183211892
209	A	0.563575638	0.376648826	0.52278301
210	A	0.303497488	0.204775403	0.717171503
211	A	0.012873301	-0.075112655	0.85430814
212	G	0.257042544	0.141632838	0.718549069
213	G	0.790193129	0.119102872	0.260008762
214	G	0.034282666	0.73769295	0.290682892
215	A	0.216627447	0.404512893	0.562860415
216	A	0.050061572	0.065602771	0.884626711
217	A	0.051242668	-0.067472874	0.598178168
218	C	0.073118544	-0.072370517	0.06237324
219	C	1.398609297	-0.138955826	0.113056105
220	A	0.606320642	-0.550063741	0.020323475
221	G	0.537907259	-0.063360683	0.006760744
222	A	0.552115786	0.10045269	0.012141116
223	G	0.89773261	-0.338769361	0.039396086
224	G	-0.132530276	0.074144534	0.744790608
225	A	0.459142295	0.516538786	0.912391931
226	G	0.882955386	0.862523739	0.977959565
227	C	0.259620294	0.117844706	0.577905725
228	U	-0.143758919	0.059608146	0.632133873
229	C	0.0947885	-0.067933931	0.079076757
230	U	0.207362306	-0.102910388	0.125745782
231	C	0.339952731	0.050214304	0.620932965
232	U	0.297542426	-0.2678854	0.150796867
233	C	0.570032527	0.078646676	0.144184473
234	G	0.601337488	0.447998782	0.742552699
235	A	0.708957282	0.859409113	0.851096193
236	C	3.958881186	-1.625727244	0.087154298
237	G	3.225037873	1.234646817	0.505644939
238	C	8.154890879	-0.250711083	0.099181132
239	A	1.83755093	-4.028022864	0.185300958
240	G	1.61010841	-0.498862469	0.18696255
241	G	1.597888561	0.612980304	0.400009763
242	A	0.423409919	0.199244035	0.656947318
243	C	0.330122982	0.244500411	0.622384165
244	U	0.236664645	0.196796436	0.873923041
245	C	0.860917621	0.428597037	0.068602138
246	G	-0.381507076	0.082546432	0.626346668
247	G	0.261824369	0.285892403	0.97122174
248	C	0.172026084	0.228099377	0.938471313
249	U	0.998686442	-0.252699417	0.30610143
250	U	2.661614746	0.465584761	0.047586371
251	G	3.702349068	0.675835661	0.060876421
252	C	3.265508831	0.403030707	0.042427037

253	U	1.775817745	0.366225258	0.030366067
254	G	1.00047042	0.093552128	0.082514772
255	A	0.905265181	0.285107524	0.129298487
256	A	0.119940006	0.012057408	0.636068287
257	G	0.204474961	0.233221391	0.762303305
258	C	-0.031386996	0.023745997	0.734776239
259	G	0.092842563	-0.049586596	0.726318929
260	C	0.009959537	0.073491367	0.693883229
261	G	-0.003559602	-0.049108355	0.86443871
262	C	1.760148163	-0.211045173	0.102509139
263	A	0.333118286	-0.035035788	0.250286426
264	C	0.091938175	-0.12274426	0.657036412
265	G	0.119388759	0.062860385	0.812914403
266	G	1.004982083	0.179960334	0.200918661
267	C	1.534847649	-0.981155433	0.049470305
268	A	0.227943352	0.143155096	0.68418317
269	A	0.392121825	0.027904946	0.057310154
270	G	0.095834771	0.188159326	0.74448894
271	A	0.221290495	0.115812608	0.831293148
272	G	1.114081778	0.096690285	0.051886138
273	G	0.279204326	-0.514744695	0.090238646
274	C	0.045660902	-1.1060851	0.328514167
275	G	0.963474804	0.742429136	0.675060464
276	A	0.899998767	0.552079225	0.338344239
277	G	1.258047315	0.465356952	0.455523157
278	G	0.222035157	1.53477164	0.552458846
279	G	7.218532847	-1.910082845	0.099243481
280	G	16.56554221	5.364500338	0.029343052
281	C	22.14131903	-0.027284132	0.070987823
282	G	1.756540532	1.512927707	0.909135506
283	G	0.262916714	-1.300485457	0.581160914
284	C	-0.512089684	-2.076000582	0.461027588
285	G	0.047918587	-1.046743774	0.474766927
286	A	0.235090079	-2.682812919	0.256368996
287	C	0.379131242	-1.851502088	0.138611317
288	U	0.207444052	-0.110178462	0.785680473
289	G	0.381638552	-0.427770104	0.539665708
290	G	1.248149273	-0.955831918	0.193467466
291	U	3.625514993	-0.375363322	0.106874121
292	G	6.10578306	-0.487926703	0.136957214
293	A	8.355994618	-0.282334681	0.051608423
294	G	8.535166758	0.730470375	0.071443178
295	U	6.963539028	-0.190315316	0.031344629
296	A	4.980006509	0.324101759	0.032796957
297	C	3.21361795	1.465943592	0.132451114
298	G	1.135181406	-0.044533537	0.237363939

299	C	0.142933348	0.194182173	0.888066933
300	C	-0.223481693	-0.188862344	0.935753503
301	A	-0.226845279	0.112075747	0.430589158
302	A	0.024264572	-0.009339555	0.853835435
303	A	0.1566111	0.086325637	0.868117573
304	A	0.311680333	0.226858938	0.821230382
305	A	0.34938455	0.232821672	0.770139315
306	U	0.728072243	-0.768414026	0.014763465
307	U	2.283606484	-0.981157905	0.005964976
308	U	1.105710783	-1.061888642	0.045448512
309	U	2.712976435	-1.537553644	0.008335003
310	G	1.822644677	0.601289049	0.018170395
311	A	0.838214806	-0.049143207	0.106352965
312	C	1.608679041	0.663522812	0.166311506
313	U	0.926012229	0.374368423	0.165276324
314	A	0.313599837	0.102041232	0.156721836
315	G	0.192923645	0.005586715	0.459710171
316	C	0.283022651	-0.140679577	0.080226334
317	G	0.826712736	0.185942342	0.077371875
318	G	2.072371352	0.269538725	0.072918565
319	A	2.121625747	-1.521484279	0.017164975
320	G	2.834143471	-0.748343439	0.02620145
321	G	4.803863528	-0.724506269	0.323161218
322	C	13.03887153	-2.863047816	0.172935639
323	U	27.38153793	-3.427033258	0.146931205
324	A	27.64334393	4.158833865	0.158935638
325	G	2.620344401	2.061923399	0.819805679
326	A	2.265741984	-0.294002252	0.040908771
327	A	1.013622225	0.663861767	0.373767673
328	G	0.286837985	-0.150806402	0.000751578
329	G	-0.007979317	-0.268882663	0.135483874
330	A	0.59739893	-0.179317925	0.025292965
331	G	1.464311096	-0.023118687	0.105195927
332	A	2.229221758	-0.348165122	0.153075806
333	G	4.050081344	-2.132653157	0.110705979
334	A	5.477882599	-3.690059162	0.089173771
335	G	8.412650825	-4.463044882	0.082439083
336	A	11.25069512	-3.100231712	0.073998835
337	U	9.357438866	-1.534160327	0.052854343
338	G	5.910655124	-3.937801831	0.024004744
339	G	5.763263532	-0.386309202	0.091591331
340	G	4.321327668	-0.588392052	0.023815968
341	U	1.345394101	4.15390546	0.1888464
342	G	-0.461184106	-1.333048311	0.330339402
343	C	0.346513899	-1.711861075	0.178897607
344	G	-1.117481079	-0.817367417	0.700557069

345	A	0.819083462	-1.04816013	0.11657657
346	G	2.937116697	1.729063587	0.495300367

Table S1

Numerical cross-linking data for RNA-Gag complexes and RNA-BSA complexes. The top 20% of values are coloured green. P value represents the statistical significance by paired t-test between RNA-Gag and RNA-BSA complexes. Those less than 0.05 are coloured purple.

Table S2

NT number	Sequence	RNA only	RNA plus Gag
36	C	0.637050249	0.379049555
37	U	0.488987558	0.481658409
38	C	-0.041579453	-0.441235226
39	U	0.48356846	0.088905762
40	C	0.269587314	-0.421904952
41	U	0.642406688	-0.070047841
42	G	0.56761227	-0.287018942
43	G	-0.21173513	0.611612142
44	C	0.337795087	-0.133728542
45	U	0.469376899	-0.001749108
46	A	0.638811542	-0.167144665
47	A	0.162128559	-0.023244208
48	C	0.138513094	0.300102776
49	U	0.210700366	0.392682394
50	A	0.347557375	0.026868654
51	G	0.135679022	-0.042337536
52	G	0.138081428	-0.432470302
53	G	-0.256740005	-0.378808916
54	A	-0.133256115	-0.227540565
55	A	-0.383212935	-0.562922695
56	C	2.491348365	-2.014766497
57	C	0.057988694	-0.063085904
58	C	0.641402114	-0.59801468
59	A	1.477603216	-0.900719522
60	C	0.207654217	-0.003851421
61	U	-0.00442723	-0.182983988
62	G	0.796343296	-0.424963149
63	C	0.324233237	0.092883136
64	U	0.026566146	-0.077434804
65	U	-0.089855696	-0.376471302
66	A	1.09207596	-0.191717549
67	A	0.082882147	-0.171066387
68	G	0.195734466	0.139091119
69	C	-0.029171134	0.166335611
70	C	-0.205239678	0.138246732
71	U	0.028522131	0.33928828
72	C	0.051915269	0.055660104
73	A	0.13862165	0.392476003
74	A	0.477119702	-0.298579845
75	U	1.135003006	-0.415907753
76	A	2.451043691	-0.134428164
77	A	1.897255373	0.447083061
78	A	1.073889663	0.25208385
79	G	0.726907598	0.306154559

80	C	0.465993639	-0.253898509
81	U	-0.100817602	-0.086595854
82	U	0.001556405	-0.543015633
83	G	0.515984468	-0.555611373
84	C	0.747660969	0.319731479
85	C	0.224344023	0.283807191
86	U	-0.011747816	0.067886457
87	U	-0.194864542	0.121832793
88	G	0.033056776	-0.065705751
89	A	-0.201112832	-0.121209131
90	G	0.059925561	0.401259363
91	U	0.083718225	0.004037417
92	G	0.364044793	0.010785671
93	C	-0.173566493	0.103311841
94	U	-0.111277398	-0.002854849
95	U	-0.075675458	0.299718478
96	C	0.24283346	0.177455284
97	A	0.253247813	0.017749289
98	A	-0.072750471	0.149500347
99	G	-0.173179182	0.162013251
100	U	-0.219695505	0.220103203
101	A	0.215409947	-0.043472231
102	G	-0.03727054	-0.07276314
103	U	-0.058072263	0.152363779
104	G	0.10274485	0.234460357
105	U	0.389723561	0.099428381
106	G	0.248573935	0.282891762
107	U	0.354517536	0.109948319
108	G	0.066183206	0.247835706
109	C	-0.103374088	0.046304901
110	C	-0.440784072	0.190791661
111	C	-0.254236917	-0.0869127
112	G	-0.080978046	-0.050923147
113	U	-0.038871002	0.050408612
114	C	0.162967496	-0.032684807
115	U	-0.079157241	-0.167203536
116	G	0.139167329	0.112261022
117	U	0.120372426	-0.068084306
118	U	0.148484996	0.278154103
119	G	0.413481809	0.200472643
120	U	-0.111123288	-0.054740239
121	G	0.293286728	0.200638541
122	U	-0.157863	-0.032085152
123	G	-0.069649574	0.151803845
124	A	-0.048890103	0.138053192
125	C	0.153338582	-0.002201395

126	U	-0.021406137	0.156004833
127	C	-0.026056451	0.203116384
128	U	-0.201640727	-0.224748382
129	G	-0.132025022	-0.194423251
130	G	0.075974309	0.164644703
131	U	-0.164083161	-0.099002249
132	A	0.107245037	0.176988003
133	A	0.605925416	0.259631883
134	C	0.307196231	0.277633939
135	U	0.066483672	0.158600255
136	A	-0.144255218	0.023002285
137	G	0.033670124	-0.338721893
138	A	-0.323832832	0.005073711
139	G	-0.116505261	0.148615857
140	A	-0.087763585	0.101501765
141	U	-0.444133007	0.178371542
142	C	0.076313339	0.630303114
143	C	-0.061444737	0.028236473
144	C	-0.059325364	0.139055423
145	U	-0.096304884	0.16157174
146	C	-0.011403767	-0.038975513
147	A	-0.224326124	-0.02311808
148	G	-0.018948742	0.20403017
149	A	-0.424616797	0.483319882
150	C	0.191596609	1.152577223
151	C	1.733266972	2.746636507
152	C	1.289321842	-2.24672029
153	U	3.185080421	-2.806335995
154	U	1.287930842	-0.276350682
155	U	-0.335453383	0.33064567
156	U	-0.228233789	0.928017942
157	A	0.089609735	-0.121769712
158	G	0.150604124	0.304432612
159	U	-0.045595937	0.09005207
160	C	-0.175661358	0.051261138
161	A	-0.302545767	-0.193718166
162	G	-0.137713365	0.162791423
163	U	-0.122233372	-0.237893033
164	G	-0.041286767	-0.194424123
165	U	-0.162400639	-0.102989045
166	G	-0.059551547	0.269486166
167	G	0.117600026	0.112818346
168	A	0.654498185	0.036461317
169	A	0.209659898	0.169070544
170	A	0.40083861	0.042911399
171	A	0.209006252	0.24561434

172	U	-0.027877077	0.121184006
173	C	0.005564457	0.158735758
174	U	0.033407007	0.074657134
175	C	-0.110339804	-0.037253508
176	U	-0.144296174	0.035351309
177	A	-0.314251264	0.070883747
178	G	0.007256332	-0.06967116
179	C	0.504441113	0.247239403
180	A	2.069984399	0.013169723
181	G	0.159334404	0.281938665
182	U	0.20299293	-0.076985488
183	G	0.28288799	-0.092440889
184	G	0.52531879	0.379011089
185	C	0.440474637	0.182219391
186	G	0.004595441	0.00260364
187	C	-0.018962828	-0.03452074
188	C	-0.108675204	-0.025952478
189	C	-0.000880808	0.003361149
190	G	-0.126212335	0.107177557
191	A	-0.00718504	0.159320874
192	A	1.005640933	0.32001853
193	C	0.25983787	0.623597132
194	A	-0.428612885	-0.287751288
195	G	-0.639567534	-0.678980151
196	G	-0.547944714	-0.164107028
197	G	-0.460775191	-0.234262143
198	A	-0.076555893	-0.409618443
199	C	0.07013283	0.098837185
200	C	0.095003605	0.089248079
201	U	0.164227231	0.204981327
202	G	0.117174518	0.224232149
203	A	0.116520866	0.212759059
204	A	-0.046170488	0.249372981
205	A	0.061598801	0.04034595
206	G	0.137527929	0.321667603
207	C	0.186895596	0.055541128
208	G	-0.152725026	-0.270953197
209	A	-0.054582142	-0.988665083
210	A	-0.062208734	0.140008759
211	A	-0.078175349	0.12167059
212	G	-0.149049894	-0.270285754
213	G	-0.099629099	0.05465522
214	G	0.230322942	0.10346673
215	A	0.17863764	0.171507991
216	A	0.280241208	0.197276194
217	A	0.430374681	0.287646029

218	C	-0.121001794	-0.200779181
219	C	-0.055429091	-0.142040558
220	A	-0.109143829	-0.301555191
221	G	0.027161454	-0.220722207
222	A	0.017744215	-0.259792879
223	G	-0.374162277	-0.524321299
224	G	-0.199765594	-0.223084047
225	A	-0.051380804	-0.337133392
226	G	-0.1182404	-0.163614563
227	C	-0.092105392	-0.113813491
228	U	-0.086290569	0.006988748
229	C	-0.044394926	0.126450993
230	U	-0.059647359	-0.091288873
231	C	-0.017121011	0.041907982
232	U	0.064784626	0.070807313
233	C	-0.212070111	-0.164853104
234	G	-0.258680439	-0.057932786
235	A	0.112537281	0.310161195
236	C	0.544201484	0.722563401
237	G	1.030782011	1.098698247
238	C	-0.404946873	0.896880089
239	A	2.817382541	0.478953178
240	G	-0.343163971	-0.054917324
241	G	0.184536656	0.299459131
242	A	0.225034329	0.267956441
243	C	-0.045420737	-0.001645393
244	U	-0.100952952	-0.080881134
245	C	-0.187989838	-0.223991605
246	G	-0.184090964	-0.353983264
247	G	-0.092072489	-0.128802633
248	C	0.118885769	0.092951336
249	U	0.085644728	-0.17780771
250	U	-0.05530216	-0.0565162
251	G	-0.090522821	-0.031669233
252	C	0.127012999	-0.268661284
253	U	0.027591804	-0.043656412
254	G	-0.122862643	-0.14827695
255	A	-0.149518444	-0.16714036
256	A	0.064830942	0.127806941
257	G	-0.005805219	-0.093303978
258	C	-0.038780463	-0.172561118
259	G	-0.108736917	-0.126751519
260	C	-0.063902603	-0.187467035
261	G	-0.025546258	-0.175887634
262	C	0.145309708	-0.008604345
263	A	0.046198529	0.103527857

264	C	-0.173024028	-0.187718621
265	G	-0.206301954	-0.315726788
266	G	0.037508762	0.002859394
267	C	0.10001179	0.086518397
268	A	-0.0861908	0.183914529
269	A	-0.185634856	-0.075442542
270	G	-0.080709246	-0.052794177
271	A	-0.162003842	-0.086919257
272	G	0.056713737	0.126381947
273	G	0.390267273	0.285888679
274	C	-0.094725401	0.036967231
275	G	-0.050724599	0.234681513
276	A	-0.149147433	0.054035756
277	G	-0.109669324	0.157422151
278	G	0.004708259	0.170643865
279	G	1.56001592	2.072683384
280	G	3.281516315	3.25848471
281	C	1.747585296	1.855480342
282	G	1.109393308	1.842920223
283	G	0.569238044	0.8170999
284	C	-0.098415664	-0.288090051
285	G	-0.008624553	0.049822939
286	A	-0.020344185	-0.04486234
287	C	-0.50800331	-0.250855949
288	U	-0.176871813	-0.001821502
289	G	0.117953485	0.307035908
290	G	0.04049718	0.792098273
291	U	0.519604945	1.102091252
292	G	0.287858328	0.764856211
293	A	1.090221937	1.164265797
294	G	0.759113724	0.932761706
295	U	0.296315693	0.674107527
296	A	0.105995095	0.078705327
297	C	0.063177053	-0.06649841
298	G	0.159841312	-0.084476448
299	C	-0.027462851	0.108844511
300	C	0.076771318	0.125947088
301	A	0.154103419	0.123509193
302	A	0.573895648	0.631724216
303	A	0.693603014	0.932403724
304	A	0.639757508	0.848357871
305	A	0.408766374	0.968305304
306	U	0.757020819	0.904820807
307	U	0.417613401	0.492405537
308	U	0.167410398	0.25384247
309	U	-0.292008077	0.202978064

310	G	-0.158259165	-0.007395611
311	A	0.124203917	0.227542948
312	C	-0.237039653	0.021435567
313	U	0.274746713	0.006445487
314	A	0.436600447	0.731809826
315	G	0.070747665	0.698796231
316	C	0.044157857	0.882904453
317	G	2.083772027	2.239742154
318	G	3.639830308	3.859161801
319	A	5.455696354	6.404923247
320	G	2.707728203	6.077691515
321	G	3.666214622	2.885454838
322	C	0.838154558	1.198358835
323	U	0.256110511	0.192887448
324	A	-0.344874166	-0.258006249
325	G	0.101491885	0.136281925
326	A	0.136012726	0.024213779
327	A	-0.191050804	0.114421607
328	G	0.365372687	0.367974887
329	G	0.588266206	0.926131778
330	A	0.923894619	2.199478289
331	G	0.738162289	2.513999001
332	A	1.00440446	3.02828447
333	G	0.474157383	2.560139188
334	A	-0.590077348	1.424002141
335	G	-2.126600282	0.000918831
336	A	-0.473152547	0.181836217
337	U	0.549155864	0.277117324
338	G	0.348695215	0.268414136
339	G	0.04881855	-0.038493947
340	G	0.067635209	0.308240736
341	U	-0.110445936	0.204966616
342	G	0.003447265	0.425068921
343	C	0.069449576	0.722215439
344	G	-0.010986325	0.144588189
345	A	-0.444522478	0.347320916
346	G	-0.107421671	0.85870771
347	A	0.635047446	0.586597958
348	G	0.848295143	1.095194767
349	C	0.44913107	0.78534208
350	G	-0.139838113	2.713123228
351	U	0.54903646	-0.418156891
352	C	1.533805768	0.735538356
353	A	1.59533239	0.157575063

Table S2: Numerical SHAPE data

Average acylation sensitivity measured for RNA-Gag and RNA-BSA complexes.
